# A Review of CXCL1 in Cardiac Fibrosis

**DOI:** 10.3389/fcvm.2021.674498

**Published:** 2021-04-28

**Authors:** Cheng-Long Wu, Ran Yin, Su-Nan Wang, Ru Ying

**Affiliations:** Department of Cardiology, The First Affiliated Hospital of Nanchang University, Nanchang, China

**Keywords:** chemokine C-X-C motif ligand-1, cardiac remodeling, cardiac fibrosis, inflammation, atrial fibrillation

## Abstract

Chemokine C-X-C motif ligand-1 (CXCL1), principally expressed in neutrophils, macrophages and epithelial cells, is a valid pro-inflammatory factor which performs an important role in mediating the infiltration of neutrophils and monocytes/macrophages. Elevated serum level of CXCL1 is considered a pro-inflammatory reaction by the organism. CXCL1 is also related to diverse organs fibrosis according to relevant studies. A growing body of evidence suggests that CXCL1 promotes the process of cardiac remodeling and fibrosis. Here, we review structure and physiological functions of CXCL1 and recent progress on the effects and mechanisms of CXCL1 in cardiac fibrosis. In addition, we explore the role of CXCL1 in the fibrosis of other organs. Besides, we probe the possibility that CXCL1 can be a therapeutic target for the treatment of cardiac fibrosis in cardiovascular diseases.

## Highlights

- In this review, we have retrospectively analyzed the role of CXCL1 in the pathological process of cardiac fibrosis.
- CXCL1 may be a potential target for the treatment of cardiac fibrosis.

## Introduction

The morbidity of heart failure (HF) is at a high level. Cardiac remodeling is a clinical process of HF, and could finally evolve into cardiac fibrosis. Cardiac fibrosis usually appears when myocardium is constantly at the stage of ischemia and hypoxia (1). It is proposed that chemokine C-X-C motif ligand-1 (CXCL1) presents significant effect during HF and ischemic cardiomyopathy (2). CXCL1 is a member of chemokines family which mediates the directional immigration of inflammatory cells, and is critical in recruiting neutrophils and monocytes/macrophages into the target position such as injured myocardium and arterial wall in CVD (2). Previous studies show that the inhibition of CXCL1 improves adverse cardiac remodeling and myocardial fibrosis thereby protects cardiac function (2, 3). Therefore, novel therapeutic method could be applied based on interference of CXCL1 to improve cardiac fibrosis. CXCL1 is also active in inflammation in other organs as a major neutrophil chemoattractant to mediate tissue injury (4, 5). However, the regulation and the mechanism of CXCL1 remain complex and obscure. More research is demanded to clarify CXCL1 and its function in CVD.

### Structure and Physiological Functions of CXCL1

**Figure 1:**
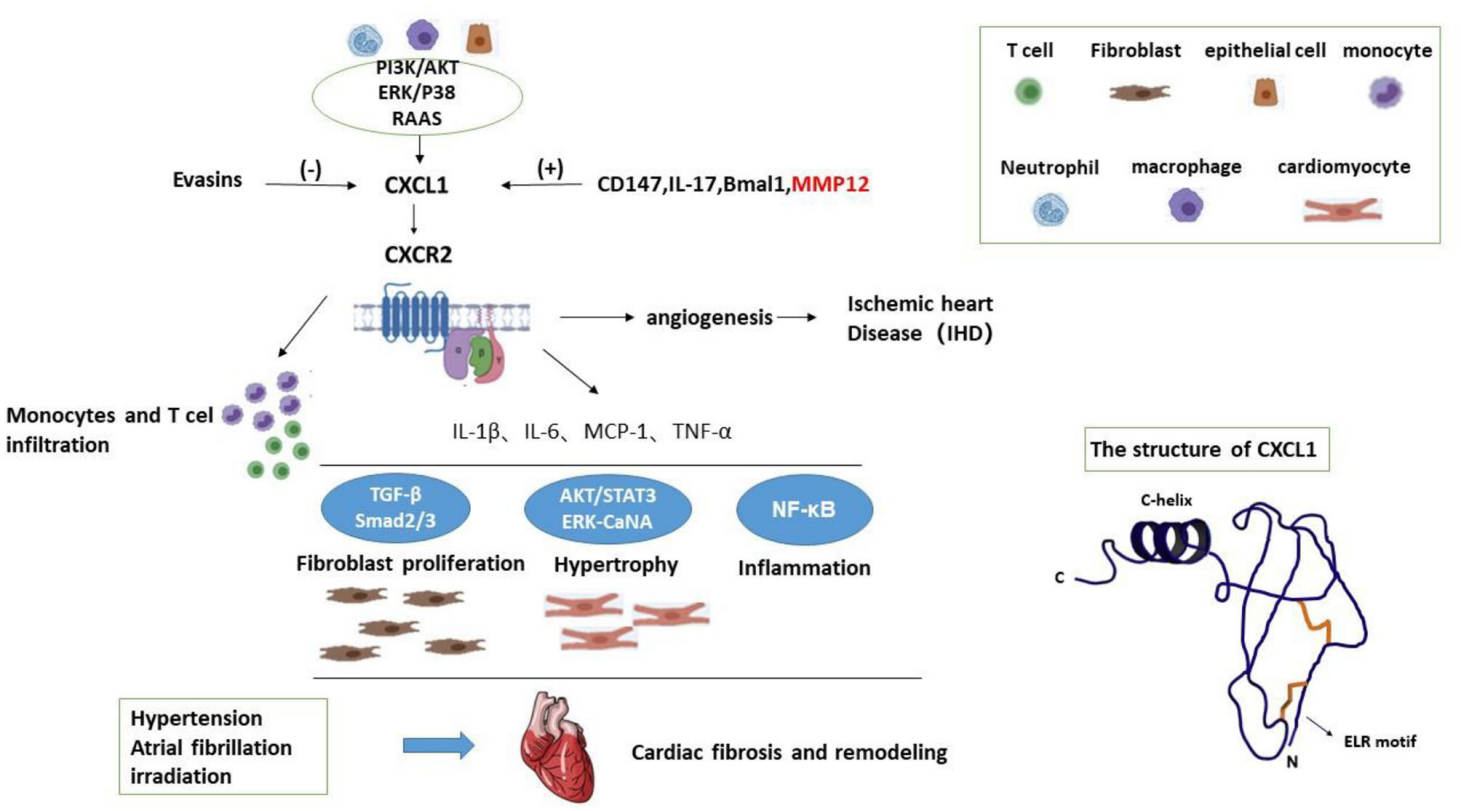
The structure of CXCL1 and working model for the mechanisms of CXCL1 in cardiac fibrosis.

CXCL1 is widely known as a strong neutrophil chemoattractant which participates in inflammation of multiple tissues (2, 6–8). It is a member of CXC superfamily which is one category of chemokines (2, 9). Chemokines are small chemoattractant molecules which recruit and activate leukocytes via specific seven-transmembrane receptors (7). They are classified into four categories according to the sequence of aminoacids related to the first 2 cysteine residues, namely CC, CXC, C, and CX3C families (7, 9, 10). The CXC family is further divided based on presence or absence of Glu-Leu-Arg sequence (ELR motif) adjacent to CXC motif (9, 11). CXCL1 is about 8 kDa (12, 13), also called growth-regulated oncogene-α (GRO-α) and keratinocyte-derived chemokine (KC) (Figure 1) (7, 11), and usually expressed in neutrophils, macrophages, and epithelial cells. CXCL1 produces its effect via its receptor CXCR2 which is mainly expressed on neutrophils and other types of cells (2).

CXCL1 exists in various tissues and produces multiple effects. As a pro-inflammatory chemokine, CXCL1 mediates acute and chronic inflammation in diverse organs thereby promotes the progress of fibrosis. The major physiology function of CXCL1 is mediating leukocyte recruitment and activation to promote inflammation and aggravate tissue injury. ELR-chemokines are valid neutrophil chemoattractants acting on G protein-coupled receptors (11). CXCL1 processes the Glu-Leu-Arg sequence and attracts neutrophils when inflammation occurs (14). Excluding inflammation, CXCL1 is also active in angiogenesis, wound healing as well as tumorogenesis depending on interaction with leukocytes, endothelial cells, and fibroblasts (7, 15). CXCL1 is related to angiogenesis during tissue remodeling (9, 16, 17). Interestingly, ELR-chemokines such as CXCL1 and CXCL2 promote vascular remodeling, whereas non-ELR CXC ligand such as CXCL4 and CXCL9 serve opposite effect (9). Besides, numerous studies indicate CXCL1 is important in tumorogenesis and progress of tumor, and CXCL1 is a promoter in evolvement of some kinds of tumor. In addition, CXCL1 widely participates in wound healing, mitosis and ischemia-reperfusion injury (18, 19).

### The Role of CXCL1 in the Development of Cardiac Fibrosis

It is believed that the main pathological characteristics of CVD are cardiac inflammation and fibrosis (20). The pathological characteristics of cardiac fibrosis could be concluded as increased interstitial fibrosis, myocyte death, and cardiac contractile dysfunction (21). Current study indicates that HF could be improved with the depression of cardiac inflammation tends to be closely relevant to the dysfunction of heart. As previously described, CXCL1 is active in mediating the infiltration of neutrophils and monocytes/ macrophages into the impaired tissues including injured myocardium and arteries. It is indicated that CXCL1 may aggravate cardiac fibrosis via pro-inflammatory effect (22). It is found that CXCL1 inhibition protects heart from Ang II-induced inflammation, hypertrophy and fibrosis (2). Mice treated with CXCL1 neutralizing antibody tend to have better cardiac function, lower level of brain natriuretic peptide and less degree of hypertrophy compared with control group. Similarly, cardiac remodeling and fibrosis are apparently alleviated through application of CXCR2 specific inhibitor. In HF patients with hypertension, the serum level of CXCL1 is obviously increased in patient with cardiac remodeling and fibrosis (2, 23). It could be concluded that CXCL1 promotes the inflammation in heart thereby accelerates the process of cardiac remodeling and myocardial fibrosis.

Atrial fibrosis is the basis of atrial fibrillation (AF). Current study indicates CXCR-2 knockout mice had significantly attenuated atrial fibrillation inducibility, atrial diameter, fibrosis, and infiltration of macrophages compared with saline-treated wild-type mice (24). Moreover, circulating blood CXCL-1 levels were higher and associated with AF in human patients compared with sinus rhythm controls (24). Besides, CXCL1 is also considered to mediate the irradiated rat cardiac fibrosis by miR-21 (25).

In sum, CXCL1 may aggravate cardiac fibrosis induced by hypertension, atrial fibrillation, post-irradiation and so on.

### Mechanisms of CXCL1 in Cardiac Fibrosis

#### CXCL1 Mediates Leukocyte Recruitment

There is growing evidence to support an important role of inflammatory cells especially monocytes/macrophages in the pathophysiology of HF (26). CXCL1 has been reported to exert a critical role in HF by regulating the recruitment of neutrophils, T lymphocytes and monocytes, especially regulating CXCR2^+^ monocytes into the heart tissues leading to cardiac remodeling and initiating and developing AF (2, 24). CXCL1 produces its effect via its receptor CXCR2 which is mainly expressed on neutrophils. CXCR2 activation plays critical roles in the recruitment of leucocytes and neutrophils, which are involved in the pathogenesis of atherosclerosis and cardiac fibrosis. In cardiovascular diseases, it is cognized that CXCL1 is vital in enlisting neutrophils and monocytes/macrophages into the impaired myocardium and artery to mediate atherosclerosis and cardiac remodeling (2). Obvious neutrophil influx mediated by CXCL1 is discovered in inflammatory sites (27).

#### CXCL1 Mediates Inflammation

The inflammation in plasma and tissue is accompanied with risen level of CXCL1 in relevant studies (28). Some researches demonstrated that the CXCL1-CXCR2 axis mediates the infiltration of monocytes into heart tissues, leading to cardiac fibrosis. CXCL1 is also active in inflammation in other organs as a major neutrophil chemoattractant to mediate tissue injury. CXCL1 may mediates inflammation by NF-κB (nuclear factor-κB) signaling, a key regulator of pro-inflammatory mediators and NOX (nicotinamide adenine dinucleotide phosphate oxidase)-2 subunit. CXCR-2 deletion reduced the activation of NF-κB signaling (24). However, whether CXCL1 transcription is regulated by NF-κB needs further study.

#### The Effect of CXCR2 on Cardiomyocyte (CM) Hypertrophy

CXCR2 was reported to promote CM hypertrophy. CXCR2 KO macrophages were co-cultured with WT neonatal rat cardiac myocytes (CMs) or fibroblasts (CFs), respectively. After 24 h of Ang II treatment, co-culture of CMs with CXCR2 KO macrophages had a significant reduction in CM size, the expression of the protein levels of p-AKT, p-ERK1/2, p-STAT3 and CaNA compared with co-culture of CMs with WT macrophages (2). Therefore, AKT, ERK1/2, STAT3 and CaNA may be the important mediators of cardiomyocyte hypertrophy.

#### CXCL1 in Cardiovascular Angiogenesis

Excluding inflammation and fibrosis, CXCL1 is also active in angiogenesis. In chronic ischemic heart disease (IHD) patients, the formation of coronary collateralization is notable. In chronic IHD, myocardial ischemia may stimulate the secretion of angiogenic chemokines such as CXCL1 to mediate the formation of coronary collateralization (9). Coronary collateralization tends to result in better cardiac function, less arrhythmias, less complications and higher survival (9). It is indicated that CXC chemokines play a vital role in the presence of coronary collaterals (9). ELR motif is structurally decisive for the physiological functions of CXC chemokines (9). The level of ELR-chemokines including CXCL1 is risen in vascular remodeling and angiogenesis. Conversely, CXC chemokines without ELR motif inhibit the progress of angiogenesis. Therefore, CXCL1 is a potent promoter of angiogenesis.

#### The Effect of CXCL1 on TGF-Smad2/3 Signaling

TGF (transforming growth factor)-Smad2/3 are the key signaling mediators of cardiac fibrosis. These researches investigated that TGF-β1, p-Smad2/3 were suppressed in CXCR-2 knockout mice, and α-SMA and collagen I also decreased (2, 24). Therefore, CXCL1 may induce cardiac fibrosis by TGF-Smad2/3 signaling.

#### Several Factors Influence the Level of CXCL1 in Cardiac Fibrosis

Many factors are known to have an impact on the serum level of CXCL1. Renin-angiotensin-aldosterone system (RAAS) is a major aspect. Angiotensin (Ang) II promotes the expression of CXCL1 (2). The inhibition of the activity of RAAS contributes to cardiac remodeling and fibrosis (2, 3). TRAF3IP2 (TRAF3 interacting protein 2T) is known as a signaling intermediate of aldosterone/salt-induced cardiac remodeling and myocardial fibrosis (3). It is discovered that the deletion of TRAF3IP2 gene significantly attenuated expression of CXCL1 (3). Meanwhile, CXCL1 mRNA level increases obviously in aldosterone-treated mice (3). Oppositely, it is indicated that Ang II modulates smoke- induced lung fibrosis and suppresses the increasing of CXCL1 (29).

IL-17 is thought a factor of adverse cardiac remodeling and fibrosis (30), and a key downstream mediator of TGF-β which is an important promote-fibrosis cytokine. It is indicated that IL-17 promotes the mRNA expression of CXCL1 and CXCL1 is a downstream gene of IL-17 (30, 31). With the neutralization of IL-17, there is a downward trend of expression of CXCL1 (30). Knockout of IL-17 gene also markedly down-regulates the level of CXCL1 in liver fibrosis (32).

Bmal1 (aryl hydrocarbon receptor nuclear translocator-like protein 1) is the gene of aryl hydrocarbon receptor nuclear translocator-like protein 1 in cardiomyocyte (33). It is presented that cardiomyocyte-specific deletion of Bmal1 significantly upgrades the transcript level of CXCL1, triggers diastolic dysfunction, extracellular matrix response, and impaired resolution of inflammation (33).

Besides, a proteolytic enzyme named MMP-9 is known to have the ability of decomposing CXCL1 (34). MMP-12 which is produced by macrophages is also related to the serum level of CXCL1 (35, 36). It is known for its function of degrading extracellular matrix, but MMP12 has several other functions. The mRNA level of CXCL1 is much higher in MMP12^−/−^ mice in infarct area of heart compared with wild type group (35). It is suggested that MMP12 inhibits infiltration of neutrophils by depression of CXCL1/CXCL2/CXCL5- CXCR2 axis (35). However, in other study, the silence of MMP12 leads to decline of CXCL1 and MMP12 is thought a contributor of secretion of CXCL1 (36). This is a contradiction we should pay attention to.

In addition to the above, a chemokine-binding protein called “Evasins” is demonstrated to have the ability of inhibiting both CC and CXC chemokine (37). Evasin-4 is proved to be relevant to the decreasing of the serum level of CXCL1 (37). CCL5 (C-C Motif Chemokine Ligand 5) is proved important in the synthesis of CXCL1 and it could be blocked by Evasin-4 (37). In addition, chemokines CC-chemokine ligand 2 (Ccl2) was related to the expression of CXCL1 in HSCs (38). In this research, Tnfr1^−/−^ /Mdr2^−/−^ mice expressed high levels of CCL2, showed significantly up-regulated hepatic gene expression of CXCL1 compared to wild type.

#### Signaling Pathway Regulating CXCL1 Expression

In addition to neutrophils, macrophages, and epithelial cells, activated hepatic stellate cells (HSCs) also release CXCL1. CD147 promotes CXCL1 expression in HSCs and CXCL1 promoted HSCs activation through autocrine (39). CD147 can bind to integrin and activates the downstream FAK/PI3K signaling pathway. CD147 overexpression induced the AKT phosphorylation; however, treating with FAK/PI3K inhibitor LY294002, CD147-induced AKT phosphorylation and CXCL1 expression were significantly inhibited. Taken together, CD147 regulates CXCL1 release in HSCs by phosphatidylinositol 3 kinase(PI3K)/protein kinase B(AKT) signaling. Therefore, PI3K/AKT signaling may be one mechanism of CXCL1 expression. A working model is illustrated in Figure 1.

MMP12 (matrix metallopeptidase 12) is proved relevant to several inflammatory diseases. Increased expression of MMP12 leads to proliferation of macrophages (36). CXCL1 is regulated by MMP12 as a pro-inflammatory chemokine (36). Knockdown of MMP12 gene reduces the expression of multiple types of cytokines including CXCL1 in animal models (36). Notably, MMP12 silencing significantly down-regulate the expression of mitogen-activated protein kinase p38(P38) and extracellular regulated kinase 1/2 (ERK1/2) and their phosphorylation (36). It is hard to say the role of the ERK/P38 MAPK signaling pathway in pro-inflammatory chemokine CXCL1 during inflammation, but it provides a new mentality to figure out CXCL1 and its regulation.

### CXCL1 in the Fibrosis of Other ORGANS

#### Lung Fibrosis

CXCL1 is connected with fibrosis of other organs in extensive research. Lung fibrosis is known as a progressive disease characterized by inflammatory infiltration and interstitial fibrosis (40, 41). Neutrophils mediate lung injury via recruitment and activation (42, 43). Neutrophil influx correlated with CXCL1 plays an important role in pulmonary fibrosis (27, 28). Inhibition of the genetic synthesis of extracellular matrix protein is accompanied with reduced level of CXCL1 (44, 45). It is suggested that IL-17 inducing chemokines promote the progress of pulmonary fibrosis (46). CXCL1 is recognized as the downstream gene regulated by IL-17 and is at a higher level in fibrosis model (46–48). IL-9 is similar to IL-17 in some experiments (49, 50). In bleomycin-induced lung fibrosis model, the release of CXCL1 is promoted by bleomycin (51, 52). Besides, CXCL1 is a novel marker for evaluating the severity of chronic obstructive pulmonary disease (28, 53). CXCL1 is also involved in airway remodeling, asthma and cystic fibrosis in lung (54–56).

#### Hepatic Fibrosis

CXCL1 is also related to hepatic fibrosis. Similar to pulmonary fibrosis, liver fibrosis also has an accumulation of extracellular matrix proteins in structure (57–59). Fibrosis in liver is usually a consequence of chronic inflammation caused by alcohol, diet and virus infections (39, 60). Neutrophils play an important role in liver injury (61). Cholestatic liver injury is accompanied with infiltration of neutrophils and upregulated expression of CXCL1 (62, 63). Decreased level of CXCL1 is observed in treatment of non-alcoholic steatohepatitis and hepatic fibrosis. Hepatic stellate cells (HSCs) are considered as a precursor of myofibroblasts in the liver and correlate with the production of extracellular matrix (64–66). CXCL1 could be secreted by HSCs in liver and CXCL1 promotes the activation of HSCs at the same time (39). HSCs stimulated by CXCL1 increase the expression of collagen type I and α-SMA (39). Therefore, CXCL1 is considered to be a promoter of hepatic fibrosis (67). Reduced expression CXCL1 is able to accelerate HSCs apoptosis thereby ameliorating liver fibrosis (68). In addition, CXCL1 performs a vital role in hepatic fibrosis induced by high cholesterol and alcohol. Blockade of CXCL1 by specific antibody reduced hepatic neutrophil infiltration and liver fibrosis in relevant study (66). Besides, M2-type macrophage mediated by CXCL1 is significantly upregulated in cirrhosis patients (69). CXCL1 could also be regulated by IL-17 in liver (70). Extracellular vesicles-derived miR-150-5p secreted by adipose- derived mesenchymal stem cells inhibits CXCL1 expression to attenuate hepatic fibrosis (5). Surprisingly, some studies suggest that CXCL1 has a protective effect against liver fibrosis and the expression of CXCL1 suppress fibrosis (58). The effect of CXCL1 in hepatic fibrosis remains further research.

#### Renal Fibrosis

All progressive chronic kidney diseases including obstructive nephropathy could result to renal fibrosis with impaired renal function (71, 72). CXCL1 acts an important role in chronic renal inflammation including obstruction as a neutrophil chemoattractant (73–75). Increased expression of CXCL1 exacerbates kidney damage in animal model (4, 76). The expression of CXCL1 gene could be controlled by NF-κB in kidney, and NF-κB is known as an indirect pro-fibrogenic factor (77). Blockade of CXCL1-CXCR2 axis efficiently alleviated renal inflammation. In addition, previous studies indicated that renal fibrosis is prompted by increased level of receptor CXCR2 (78). Macrophages mediated by CXCL1 are correlated with collagen formation, extracellular matrix deposition and the degree of renal dysfunction in experimental animal models (79). CXCL1 is also relevant to the fibrosis of intra-allograft in some cases (80).

#### Other Organs

CXCL1 is also related to the fibrosis in pancreas and biliary atresia (81, 82). What's more, fibrosis after autoimmune thyroiditis is also supposed relevant to CXCL1 (83).

## Conclusion

CXCL1 is a potent neutrophil chemoattractant, which could be secreted by macrophages, fibroblasts, keratinocytes, and epithelial cells (84). It is involved in diverse tissue inflammations, fibrosis, tumor, angiogenesis in various tissues. In CVD, CXCL1 plays an important role in cardiac fibrosis especially induced by hypertension, atrial fibrillation, post-irradiation. Therefore, CXCL1 is a promising target and antagonism of CXCL1 or CXCR2 is a novel therapy for treatment of cardiac fibrosis. However, whether cardiac fibrosis induced by other causes (such as lone AF, aortic coarctation and myocardial infarction) is associated with CXCL1 is not clear. Hence, more researches need to conduct to ascertain the roles and relevant mechanisms of CXCL1 in these cardiac fibrosis. Actually, we have been doing related research, and we found that the oxidative stress related pathway might contribute to the effect of CXCL1 in cardiac fibrosis. However, we need more research. With the aid of further researches of CXCL1, it will help cardiologists devise more reasonable therapeutic plans and improve the prognosis of CVD patients.

## Data Availability Statement

The raw data supporting the conclusions of this article will be made available by the authors, without undue reservation.

## Author Contributions

C-LW: the main writer. RYin: reference collection and screening. S-NW: typography and format modification. RYing: manuscript design, modification, and submission. All authors contributed to the article and approved the submitted version.

## Conflict of Interest

The authors declare that the research was conducted in the absence of any commercial or financial relationships that could be construed as a potential conflict of interest.

## Abbreviations

CVD: cardiovascular disease
HF: heart failure
Ang: angiotensin
IL: Interleukin
HSCs: hepatic stellate cells
IHD: ischemic heart disease
RAAS: renin-angiotensin-aldosterone system
MMP-12: matrix metallopeptidase 12
LPS: lipopolysaccharide
CCL: C-C Motif Chemokine Ligand.
